# Factors associated with the identification and well-being of hidden and confirmed carers: a dyad analysis

**DOI:** 10.1093/geronb/gbaf129

**Published:** 2025-07-04

**Authors:** Elise Whitley, Michaela Benzeval

**Affiliations:** MRC/CSO Social and Public Health Sciences Unit, University of Glasgow, Glasgow, United Kingdom; Institute for Social and Economic Research, University of Essex, Colchester, United Kingdom; School of Health and Wellbeing, University of Glasgow, Glasgow, United Kingdom

**Keywords:** carer, caregiver, identity, hidden carer

## Abstract

**Objectives:**

Growing numbers of older people living with disability or long-term conditions are being cared for by family members and friends on an informal, unpaid basis. “Hidden carers,” who provide care to another person but either do not recognize their role or consciously reject it, are overlooked by policymakers and services that rely on self-identification.

**Methods:**

We compare reports of informal care provision and receipt between dyads of household members in Understanding Society to identify hidden (*N* = 5,615) vs. confirmed (*N* = 3,129) informal carers and explore their characteristics and well-being outcomes.

**Results:**

Hidden carers tended to support someone with fewer limitations or difficulties, but were more likely to be in need/in receipt of informal care themselves. Informal carers who were receiving formal care or caring for someone who had more contact with health and care professionals were more likely to identify their caring role; however, the same was not true for their own contact with health services. Compared with confirmed carers, hidden carers had better mental health and greater life satisfaction, and, among couples, were happier with their relationship with their partner for whom they cared.

**Discussion:**

Identification of informal carers is a priority for policymakers, but self-identification is complex, with potentially contradictory barriers. Focusing on caring activities rather than relying on self-identification may be a more acceptable means of identifying informal carers who are in need of support.

Populations are ageing, with increasing numbers of older people living with disabilities or long-term health conditions requiring care, the majority of which is delivered by family and friends on an informal, unpaid basis (WHO Regional Office for Europe, 2022; World Health Organization, 2021a). Informal caring is commonly reported as stressful (Carers UK, 2022; Hirst, 2005; Schulz et al., 2020; Schulz & Sherwood, 2008), with high levels of unpredictability, uncontrollability and vigilance, and carers often have worse mental and physical health when compared to non-carers (Bom et al., 2018; Cuijpers, 2005; Hirst, 2005; Pinquart & Sörensen, 2003; Schoenmakers et al., 2010; Schulz et al., 2020; Schulz & Sherwood, 2008; Smith et al., 2014; Stansfeld et al., 2014). However, benefits of informal caring are also reported (Roth et al., 2015; Schulz et al., 2020; Schulz & Sherwood, 2008), including fulfilment, personal growth, and confidence that the care recipient is receiving good quality care.

World Health Organization initiatives on integrating long-term care highlight the importance of “robust data” (WHO Regional Office for Europe, 2022; World Health Organization, 2021b), which depends on informal carers’ self-identification with the role. However, entry into informal caregiving is usually a gradual process rather than a discrete event, and people may only self-identify as a carer once they are providing a broad range of care (Schulz et al., 2020). Consistent with this, UK statistics indicate that half of unpaid caregivers take over a year to recognize their role and more than a third take over 3 years (Carers UK, 2022). Research with dyads of informal carers and care recipients reveals high levels of disagreement in reports of care provided and received (Rodrigues et al., 2024; Rutherford & Bu, 2018; Urwin et al., 2021), which may also vary from day to day (Godfrey et al., 2018). There is also discordancy between informal carers’ self-identification with the role and their self-reported caring activities (AARP, 2001; Hughes et al., 2013; Urwin et al., 2022).

Interviews with informal carers highlight barriers to identification (Eifert et al., 2015; Knowles et al., 2016), including relationship and gender norming, and the conscious rejection of the “carer” label in order to maintain relationships with the care recipient and protect them from stigmatizing “in need” labelling (Carers UK, 2022; Carduff et al., 2014; Eifert et al., 2015; Hughes et al., 2013; Knowles et al., 2016). However, interviewees also report that self-identification may be prompted through interactions with others (Carers UK, 2023; Eifert et al., 2015) including family members, other carers, and, in particular, professionals involved in the recipient’s care (Carduff et al., 2014; Carers UK, 2023; Corden & Hirst, 2011; Knowles et al., 2016; Molyneaux et al., 2011; O'Connor, 2007).

“Hidden carers,” who do not identify with the role, are overlooked by policy makers and services that rely on self-identification (Eifert et al., 2015; Schulz et al., 2020). A handful of studies have explored predictors of self-identification in (primarily spousal) dyads and suggest that hidden carers live in larger households and have been caring for less time, for fewer hours, and for someone with fewer health conditions/limitations (Rodrigues et al., 2024; Rutherford & Bu, 2018; Urwin et al., 2021). This lower caring burden might suggest more favourable outcomes in hidden carers, but survey data demonstrates that they are also less likely to access support (Eifert et al., 2015; Carers UK, 2023), which may have a detrimental impact. Comparisons of outcomes in hidden vs. confirmed carers are rare, with only a single study, which reported no clear differences in terms of self-rated health or depression (Rodrigues et al., 2024). Given the complexity of the identification process and the potentially mixed consequences of caring self-identity, it is important to investigate more fully the predictors and sequelae of hidden caring.

We extend previous work using data from a large population household survey to explore determinants and well-being outcomes of hidden caring in dyads of (potential) carers and care recipients. In particular, we answer the following questions: (1) what characteristics are associated with being a hidden vs. confirmed carer; (2) does contact with health and care professionals influence carer self-identification; and (3) what is the impact of carer self-identification on mental health, life satisfaction, and quality of relationship with the care recipient.

## Method

Analyses use data from *Understanding Society*, the UK Household Longitudinal Study (UKHLS) (Benzeval et al., 2023; Lynn et al., 2023), a longitudinal survey of over 40,000 households in England, Scotland, Wales and Northern Ireland based on a probability sample drawn in 2009. All household members are contacted annually to collect information on changes to their circumstances. The University of Essex Ethics Committee has approved all data collection on the Understanding Society study. The current analyses focus on households comprising two or more adults (aged 16+), in which all eligible members responded to ensure that all caring relationships within the household were captured.

### Identification of hidden/confirmed carers

At every wave, adults are asked: “Is there anyone living with you who is sick, disabled, or elderly whom you look after or give special help to (for example, a sick, disabled, or elderly relative, husband, wife, or friend, etc)?” Those who said yes were identified as informal carers and follow-up questions were asked about which household member(s) they supported. In waves 7 (2015–2017), 9 (2017–2019), 11 (2019–2021), and 13 (2021–2023), respondents aged 65+ who reported needing help with one or more activities of daily living (ADL) or instrumental activities of daily living (IADL) were asked: “In the last month who has helped you with [ADL/IADL]?” with follow-up questions identifying the household member(s) who provided this help.

The current analyses use pooled cross-sectional data from these waves and are restricted to households where at least one member was in need (aged 65+ with ADL/IADL limitations) and identified another household member as providing care. All household dyads were examined to compare accounts of care receipt and provision. Confirmed carers were identified from dyads in which care receipt and provision were confirmed by both household members. Hidden carers were identified from dyads in which one member identified the other as providing care, but this was not confirmed by the person identified. Dyads in which both members agreed that there was no care or where the recipient did not confirm caring provision were omitted from the current analyses.

### Determinants of hidden caring

Potential determinants were based on previous literature (Corden & Hirst, 2011; Rodrigues et al., 2024; Rutherford & Bu, 2018; Urwin et al., 2021, 2022). Care recipient characteristics included age in years, sex, number of ADL/IADL limitations, and presence (yes/no) of self-reported sensory (hearing or sight), physical (mobility, lifting and carrying, dexterity, continence, physical coordination, or physical care), or neurological (communication and speech, memory and learning, or recognizing danger) difficulties. Carer characteristics included age, sex, relationship with care recipient (spouse, child, or other), any ADL/IADL limitations (yes/no), and, if so, whether they received care from another household member (yes/no).

The role of professionals in caring self-identification considered contact with both care recipients and (potential) carers. In each case, contact with health professionals was based on any vs. no GP contact, outpatient contact, or inpatient stays in the previous year. For those with ADL/IADL limitations (all care recipients and a subgroup of potential carers) contact with formal care services was also considered, comparing those with and without support from: home care worker, home help, personal assistant, reablement/intermediate care staff, occupational therapist, physiotherapist, nurse, voluntary helper, warden/sheltered housing manager, cleaner, or council handyman.

### Potential outcomes of hidden caring

Hidden and confirmed carers’ mental health was assessed using the 12-item General Health Questionnaire (GHQ-12) (Goldberg et al., 1997), with scores below the mean (in this case 3) indicating good mental health (Jokela et al., 2021). Satisfaction with life was based on responses to the question: “How dissatisfied or satisfied you are with your life overall,” dichotomized as satisfied vs. dissatisfied or neither satisfied, or dissatisfied. For spousal dyads, relationship satisfaction and cohesion were assessed using the Revised Dyadic Adjustment Scale (RDAS) (Busby et al., 1995), with an additional question rating the overall happiness of their relationship. Responses were dichotomized as satisfaction/cohesion above vs. below the median, and relationship happiness was rated as happy or perfect vs. unhappy.

### Statistical analyses

Analyses focused on household members who were identified as providing care by a person in need. In most dyads (93%), this person was uniquely identified. In the remainder, there was caring reciprocity (both dyad members were care recipients and cared for the other), and in these, one person was selected at random as the care recipient and the other as the carer. Logistic regression, carried out in Stata v18.0 (StataCorp, 2023), was used to provide odds ratios (ORs) for two groups of models. First, for being a hidden vs. confirmed carer according to care recipient and respondents’ characteristics and contact with professionals, unadjusted and adjusted for care recipient characteristics. Second, for better mental health, life satisfaction, and relationship quality according to hidden vs. confirmed caring status, with and without adjustment for care recipient characteristics.

## Results

In total, 5,014 households were identified as having someone “in need,” having complete interviews for all adult members, and containing at least one person aged 65+ with at least one ADL/IADL limitation. Of these, 4,062 (81%) households (8,744 individuals) included at least one person in need of care who identified another household member as providing help. Individuals in the analytical sample were very similar to all individuals within “in need” households (Table 1). Almost two-thirds of individuals (*N* = 5,615) included in the analyses were hidden rather than confirmed (*N* = 3,129) carers.

**Table 1.** Characteristics of analytical sample compared with “in need” household members.

Characteristic	Analytical sample (*N* = 8,744)	All “in need” household members (*N* = 10,733)
% male	48.5	48.6
% aged 65+	83.1	83.3
% sensory difficulties	22.5	21.5
% physical difficulties	54.1	50.3
% neurological difficulties	19.5	18.3
Mean number of ADL/IADL limitations	1.9	1.7
% hidden carer	64.2	–

*Note.* ADL = activities of daily living; IADL = instrumental activities of daily living.

ORs for being a hidden vs. confirmed carer are presented in Table 2. Hidden carers were less likely to be providing support to individuals with more difficulties or ADL/IADL limitations. In models adjusted for care recipient characteristics, carers who had any ADL/IADL limitations and, in particular, reported receiving care from another household member were markedly more likely to be hidden carers (OR [95% CI] for any vs. no ADL/IADL limitations: 2.31 [1.88, 2.84]; OR [95% CI] for receiving care from another household member: 5.92 [4.26, 8.24]). Individuals providing care to household members with more professional contact were markedly less likely to be hidden carers, even after adjusting for care recipient characteristics (e.g., OR [95% CI] for care recipient GP contact in the last year: 0.37 [0.27, 0.50]). However, in contrast, carers’ own contact with health professionals had no marked association with their odds of being a hidden carer, although those who received formal care were less likely to be hidden carers (0.43 [0.29, 0.64]).

**Table 2.** OR (95% CI) for being a hidden vs. confirmed carer according to (potential) carer and care-recipient characteristics.

Characteristic	OR (95% CI) for being hidden vs confirmed carer	
	Unadjusted	Adjusted^a^
*Care recipient characteristics*		
Age (1 year increase)	0.95 (0.94, 0.96)	-
Female	0.95 (0.84, 1.08)	-
Sensory difficulties	0.59 (0.51, 0.68)	-
Physical difficulties	0.15 (0.13, 0.18)	-
Neurological difficulties	0.40 (0.34, 0.46)	-
N ADL/IADL limitations (1 additional)	0.56 (0.54, 0.58)	-
*(Potential) carer characteristics*		
Age (1 year increase)	1.01 (1.00, 1.01)	1.00 (0.99, 1.01)
Female	0.94 (0.83, 1.07)	0.83 (0.61, 1.14)
Relationship with care recipient		
Child (vs spouse)	0.50 (0.41, 0.61)	1.05 (0.80, 1.38)
Other (vs spouse)	0.74 (0.50, 1.11)	1.25 (0.74, 2.10)
Any ADL/IADL limitations	1.68 (1.45, 1.96)	2.31 (1.88, 2.84)
Also receive care from household member	6.18 4.66, 8.19)	5.92 (4.26, 8.24)
*Care recipient contact with professionals*		
GP contact in last year	0.36 (0.28, 0.45)	0.37 (0.27, 0.50)
Outpatient contact in last year	0.40 (0.35, 0.47)	0.47 (0.39, 0.58)
Inpatient stay in last year	0.36 (0.31, 0.42)	0.51 (0.41, 0.62)
Receive formal care	0.46 (0.39, 0.53)	0.78 (0.64, 0.96)
*(Potential) carer contact with professionals*		
GP contact in last year	0.97 (0.84, 1.14)	1.04 (0.85, 1.27)
Outpatient contact in last year	1.07 (0.94, 1.22)	1.10 (0.92, 1.30)
Inpatient stay in last year	1.16 (0.93, 1.43)	1.10 (0.83, 1.46)
Receive formal care	0.45 (0.34, 0.59)	0.43 (0.29, 0.64)

*Note*. ADL = activities of daily living; CI = confidence interval; GP = general practitioner; IADL = instrumental activities of daily living; OR = odds ratio.

a Adjusted for care recipient age, sex, difficulties, and individual ADL/IADL limitations.

Comparing the well-being of hidden vs. confirmed carers (Table 3), hidden carers had better mental health (OR (95% CI) for GHQ-12 score <3: 1.31 [1.06, 1.62]) and were more likely to report being satisfied with their life overall (1.69 [1.45, 1.98]). Among spousal carers, relationship satisfaction and cohesion were greater in hidden vs. confirmed carers (unadjusted OR [95% CI] for above median satisfaction and cohesion: 1.18 [1.02, 1.36] and 1.33 [1.15, 1.53] respectively), although the association with cohesion was explained by adjustment for care recipient characteristics. Hidden carers were also more likely than confirmed carers to be happy with their relationship, with no attenuation after adjustment for care recipient characteristics (1.50 [1.10, 2.04]).

**Table 3.** OR (95% CI) for better relationship quality, mental health, and life satisfaction according to hidden versus confirmed carer status.

Relationship, mental health, life satisfaction measure	OR (95% CI) for positive outcome	
	Unadjusted	Adjusted^a^
*All dyads*		
GHQ-12 (better mental health (<3))	1.45 (1.23, 1.71)	1.31 (1.06, 1.62)
Life satisfaction (satisfied vs. not satisfied)	1.69 (1.45, 1.98)	1.31 (1.07, 1.60)
*Spousal dyads*		
RDAS satisfaction (above median)	1.18 (1.02, 1.36)	1.19 (0.99, 1.44)
RDAS cohesion (above median)	1.33 (1.15, 1.53)	1.00 (0.83, 1.21)
Happiness with relationship (happy vs. not happy)	1.48 (1.18, 1.86)	1.50 (1.10, 2.04)

*Note*. ADL = activities of daily living; CI = confidence intervals; GHQ-12 = 12-item general health questionnaire; IADL = instrumental activities of daily living; OR = odds ratio; RDAS = revised dyadic adjustment scale.

a Adjusted for care recipient age, sex, difficulties, and ADL/IADL limitations.

## Discussion

Ageing populations are leading to an increasing reliance on informal care, but evidence suggests that many carers do not self-identify with the role. At an individual level, hidden carers may miss out on financial and respite support to which they are entitled (Carers UK, 2023) while, at a population level, their omission from official statistics and surveys is a significant barrier to initiatives aimed at integrating informal and formal care (WHO Regional Office for Europe, 2022; World Health Organization, 2021b).

We confirm previous findings (Urwin et al., 2021) that hidden carers tend to care for individuals with fewer limitations or difficulties (Rutherford & Bu, 2018; Urwin et al., 2021). However, after adjusting for care recipient characteristics, we also find that carers who themselves are in need of or in receipt of informal care are markedly less likely to self-identify as a carer, potentially indicating that the reciprocity means that caring activities are regarded simply as everyday tasks. The role of professionals in encouraging carer recognition is identified in qualitative research (Carduff et al., 2014; Corden & Hirst, 2011; Knowles et al., 2016; Molyneaux et al., 2011; O'Connor, 2007) and survey data (Carers UK, 2023), with National Institute for Health and Care Excellence guidance (National Institute for Health and Care Excellence, 2020) stating that practitioners should “use every opportunity to identify carers.” Our results indicate that the carers of people with more professional contact are more likely to recognize their role, potentially reflecting greater morbidity in these care recipients, but similar results were not observed for carers’ own contact with health professionals. However, while carers in need of care were more likely to be hidden, those in receipt of formal care were more likely to recognize their caring role, suggesting that professionals in this context may be important in encouraging self-identification.

Hidden carers may miss out on benefits or support to which they are entitled and are often presented as a high-risk, disadvantaged group (Carers UK, 2022, 2023; Schulz et al., 2020). However, in our data, hidden carers had better mental health and greater life satisfaction than confirmed carers, even after adjustment for care recipient difficulties and limitations. Interviews indicate that individuals may reject the caring label in order to preserve their relationship with the care recipient ­(Eifert et al., 2015; Hughes et al., 2013; Knowles et al., 2016; Molyneaux et al., 2011) and, consistent with this, our hidden spousal carers were more satisfied with their relationship than those who self-identified as a carer, although the association with relationship cohesion was attenuated by adjustment for care recipient characteristics, potentially reflecting limitations to shared activities with a sicker/more disabled individual.

We have, unusually, compared household members’ reports of caring provision and receipt in order to identify confirmed and hidden carers in a large population-based survey. We consider a broader range of carer and care recipient characteristics than previous research, in particular contact with professionals, and we compare well-being outcomes of hidden and confirmed carers, which has not been widely done before. However, there are also limitations to consider. Care receipt questions were only asked of household members aged 65+ with one or more ADL/IADL limitations and, while this approach will identify many older people in need of care, there will be some who were missed. In particular, we did not consider carers who supported someone outside their household, and, as these individuals may be at an earlier stage in their caring journey, hidden carers in this context are likely to have been underrepresented. Additionally, although older adults are the most common recipients of informal care (Schulz et al., 2020), there are groups missing from our analyses, such as younger adults needing care, and people with dementia whose condition does not impact ADL/IADL activities but nevertheless require care. Our results, particularly analysis of outcomes, are therefore potentially only relevant to co-resident caring for older individuals with ADL/IADL limitations. Previous work highlights the importance of care recipient difficulties and limitations in determining hidden vs. confirmed caring status. If we have not taken sufficient account of these factors, this might explain our associations, although extensive adjustment for care recipient characteristics had a limited impact on results, making this explanation unlikely. Finally, we cannot exclude the possibility that individuals who were more satisfied with their life and relationship were less likely to identify as a carer as a result. However, these results are consistent with qualitative work highlighting reluctance to identify as a carer in order to preserve relationship identity and quality (Molyneaux et al., 2011).

## Conclusion

The identification of informal carers is important in order to support carers themselves and also the people to whom they provide support. However, carer self-identification is a complex process with potentially contradictory barriers, and carers who do not recognize the role may have better outcomes. Focusing on caring activities rather than individual carer and care recipient roles may offer a more acceptable route to identifying individuals providing and in need of support, particularly when caring is reciprocal. In everyday clinical settings, discussing informal support received by people with health conditions may help identify and provide support for their carers. For policymakers, understanding the full extent of informal caring is important to formulate relevant financial and respite policies. At a societal level, considering how to counter the negative connotations of being in need or caring may help reduce the lack of identification of the caring role and/or its detrimental impact on well-being and relationship quality.

## Data Availability

Data are made available under license via the UK Data Service (https://beta.ukdataservice.ac.uk/datacatalogue/series/series?id=2000053).
